# Contribution of conspecific negative density dependence to species diversity is increasing towards low environmental limitation in Japanese forests

**DOI:** 10.1038/s41598-021-98025-5

**Published:** 2021-09-21

**Authors:** Pavel Fibich, Masae I. Ishihara, Satoshi N. Suzuki, Jiří Doležal, Jan Altman

**Affiliations:** 1 Institute of Botany of the Czech Academy of Sciences, Průhonice, Czech Republic; 2 Department of Botany, Faculty of Science, University of South Bohemia, České Budějovice, Czech Republic; 3 Field Science Education and Research Center, Ashiu Forest Research Station, Kyoto University, Kyoto, Japan; 4 Graduate School of Agricultural and Life Sciences, The University of Tokyo Hokkaido Forest, The University of Tokyo, Furano, Japan

**Keywords:** Forest ecology, Ecology, Biodiversity, Plant sciences, Plant ecology

## Abstract

Species coexistence is a result of biotic interactions, environmental and historical conditions. The Janzen-Connell hypothesis assumes that conspecific negative density dependence (CNDD) is one of the local processes maintaining high species diversity by decreasing population growth rates at high densities. However, the contribution of CNDD to species richness variation across environmental gradients remains unclear. In 32 large forest plots all over the Japanese archipelago covering > 40,000 individual trees of > 300 species and based on size distributions, we analysed the strength of CNDD of individual species and its contribution to species number and diversity across altitude, mean annual temperature, mean annual precipitation and maximum snow depth gradients. The strength of CNDD was increasing towards low altitudes and high tree species number and diversity. The effect of CNDD on species number was changing across altitude, temperature and snow depth gradients and their combined effects contributed 11–18% of the overall explained variance. Our results suggest that CNDD can work as a mechanism structuring forest communities in the Japanese archipelago. Strong CNDD was observed to be connected with high species diversity under low environmental limitations where local biotic interactions are expected to be stronger than in niche-based community assemblies under high environmental filtering.

## Introduction

Understanding drivers of species diversity has been a major challenge in both theoretical and applied ecology. Species coexistence is a result of biotic interactions as well as environmental conditions^1^. Negative density-dependent interactions, together with specialized natural enemies, are one of the processes expected to maintain high species diversity^2^. Conspecific negative density dependence (CNDD) is the process that decreases population growth rates at high densities, because of natural enemies and competition for resources, and therefore favors locally less common species over common ones^3^. The Janzen-Connell hypothesis assumes that CNDD is the key process responsible for reduced recruitment near conspecific adults, creating space available for other species and therefore enhancing local diversity^4^. Moreover, negative effects can be more complex, and high densities of heterospecifics or pressure from generalist natural enemies can also reduce the growth rate of a given species^5^. Thus, CNDD is expected to maintain diversity only when it is stronger than the negative effects from heterospecific densities. However, exactly how CNDD affects species diversity still remains unclear^6,7^.

Decreasing strength of CNDD was recently observed from the tropical to temperate forests, followed by decreasing species richness^7^. CNDD was also stronger for rare species in the tropics than in the temperate latitudes. Strong CNDD can reduce extinction risk by making the population dynamics of rare species more stable and therefore hold their high numbers^7–9^. This supports the persistence of high numbers of rare species in the tropics. Several mechanisms were suggested to explain the shift in strength of CNDD across latitudes (e.g. stronger intra-specific competition or pressure from natural enemies in the tropics than in the temperate latitudes, strong dispersal limitation for rare species and their enemies in the tropics). On the other hand, LaManna et al^7^ computed CNDD from a single census data by quadrate-based approach and their method started a debate leading to a discussion about the correctness and limitations of such results^10,11^, and about the effect of aggregation on CNDD and generally about the detectability of CNDD even in studies with re-censuses during the time^12^. Nevertheless, LaManna et al^13–15^ supported their previous results of CNDD along the latitudes by several additional methods (e.g. new null models).

Among other factors (e.g. evolutional history, space, biotic interactions), the climate is expected to directly affect patterns of species diversity^16^ and may also have indirect influence by altering the composition of natural enemies, their effects on host species and also the strength of intraspecific competition^17^. For example, Janzen^18^ suggested that species in the tropics experience a narrower range of temperature than those in temperate latitudes leading to higher specialization in the tropics. In addition to such local processes (e.g. biotic interactions), regional effects (i.e. evolutional, geographic and geology history) also influence global patterns of tree diversity^16,19,20^. Generally, the climate and regional effects are setting limits as to what species could occupy sites if all other factors were equal. From the climatic variables, mostly used determinants of plant distribution, species richness and productivity are measures of temperature and water availability^16,21–23^. Tree species richness is mostly positively correlated with temperature and precipitation, and the decrease or mid-peak of species richness is often observed along an elevation gradient^24,25^. The elevation is tightly correlated with temperature, but could have a more complex relationship with precipitation, even though elevation and precipitation are mostly positively correlated^26^. The strength of negative density dependence and species richness increased with resource availability in the temperate forest^27^. On the global scale, the strength of CNDD in woody-plant species increases with precipitation and productivity^6,20^. Climate effects (temperature and precipitation) were observed to be less important for the recruitment than density dependence^28^. On the other hand, larger trees often respond stronger to abiotic habitat characteristics than biotic interactions such as neighborhood competition^29^. Stronger CNDD is expected to be the effect of stronger intraspecific competition, increased virulence, herbivore pressure and/or host-specific pathogens^30,31^. However, to what extend CNDD contributes to species richness variation across a wider resource or environmental gradients remains to be tested.

Both historical (e.g. geographical isolation, evolutional history) and ecological factors contributed to the current high diversity of trees in temperate forests in Japan^32–34^. The regional climate ranges from mesic to humid in Japan. Therefore, there is low limitation by precipitation and the distribution of tree species is generally controlled by temperature^35,36^. For example, forest productivity was mainly controlled by the temperature gradient, but not by precipitation in Japanese forests^37^. Also, directional changes in the abundance of tree species and functional types were observed along the temperature gradient in Japan^38^. Higher recruitment, lower mortality and higher population growth were observed at the colder range boundaries, which might be due to climate changes or past disturbances. Snow cover, though, is also expected to strongly affect plant distribution and recruitment in Japan^39–41^, e.g. *Fagus crenata* predominates widely in the cool-temperate zone, especially in snow-rich sites bordering the Sea of Japan^42,43^.

In the present study, we examined the strength of CNDD along several environmental gradients, and their combined effect on species richness in 32 large (0.8–1.2 ha) stem-mapped forest plots in Japan. Specifically, we explored three hypotheses: (1) the strength of CNDD is increasing along environmental gradients towards low environmental limitation, (2) species diversity is correlated with the strength of CNDD, and (3) the effect of CNDD on species diversity is affected by environmental gradients. We hypothesized that CNDD is not stable along environmental gradients (i.e. across temperature or snow depth gradients as the key drivers of tree species distribution in Japan^36,39^). Specifically, a tight correlation of species diversity with the strength of CNDD is expected, e.g. due to a similar response to environmental gradients^16^. On the other hand, we expected the effect of CNDD on species diversity to be affected by environmental gradients, because the increasing strength of environmental limitation can reduce the relative importance of biotic interactions. Although the number of studies on CNDD impacts across local small-scale environmental gradients is increasing^27,29,44^, the CNDD studies across wide environmental gradients in the temperate forest are still lacking (but see seedling studies^6,28^). Our study is the first which focuses on a combination of locally computed CNDD and its contribution to species diversity along multiple environmental gradients in highly diverse Japanese forests.

## Methods

### Dataset

**Figure 1:**
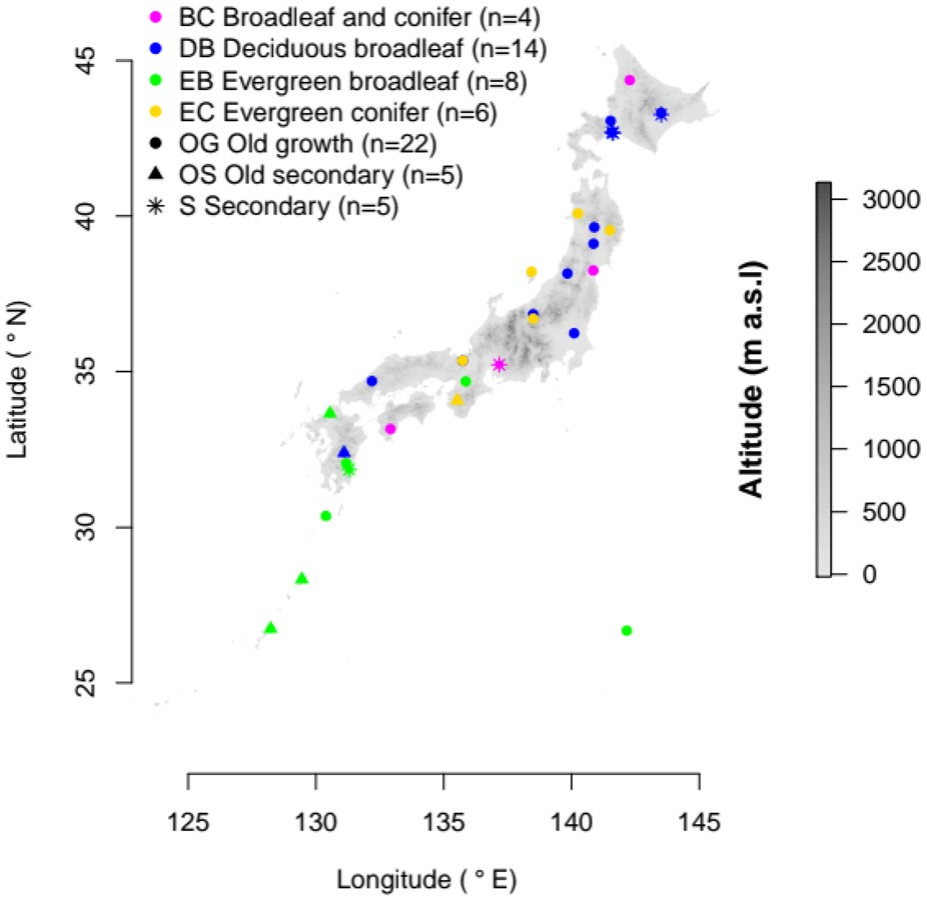
Altitude map of studied forest sites according forest types and succession stage categories in Japan (altitude drawn in R^52^ version 3.6.1 based on data from the raster package version 3.4-5).

We analysed data from 32 permanent forest plots (0.8 ha to 1.2 ha) across Japan (Fig. 1; Table S1), that cover a broad range of environmental gradients (further details^45^). For each plot, altitude (range 33–1730 m a.s.l.), mean annual temperature (range 3.5–21.3 °C, hereafter temperature), mean annual precipitation (range 868–3677 mm, hereafter precipitation) and mean maximum snow depth (range 0–1.17 m, hereafter snow depth) were extracted from the 1 km grid resolution database Mesh Climate Data 2000, distributed by the Japan Meteorological Agency (2002) for years 1971 to 2000. Snow depth was ln(x + 1) transformed, because of high skewness. The latitudinal gradient is not presented, because it was closely correlated with temperature (r = − 0.83, P < 0.001). Trees’ girth at breast height (GBH) and species identity were recorded if their GBH ≥ 15 cm (corresponding to DBH ≥ 4.8 cm) measured between 2005 and 2009. Overall we analyzed 42,359 trees from 312 unique species. To characterize species diversity, the number of species (S), Shannon index of diversity (H = − Σ*p*_*i*_ ln *p*_*i*_, where *p*_*i*_ is the proportion of individuals of species *i* from all individuals) and Pielou's evenness (e = H/ln S) for all plots were calculated (natural logarithm was applied in all computations). The forests were divided according to: 1) the prevalent functional types of trees into evergreen broad-leaved (EB), deciduous broad-leaved (DB), broad-leaved and conifer mixed (BC) and evergreen conifer (EC); and 2) succession stage category into old-growth (OG), old secondary (OS) and secondary forests (S). Secondary forests were 60–100 years from the last major disturbance, while for old-growth and old-secondary forests was this period even longer. The effect of typhoons (the dominant disturbance agent) frequency and intensity was checked and no correlations with the plots species numbers were observed.

### Analyses of conspecific negative density dependence (CNDD)

CNDD was computed from the last census of each plot in two quadrat sizes of 10-by-10 m and 20-by-20 m, because interactions between trees and conspecific density strongly decay between 10 and 20 m distances^46^. The re-census data were not used because just 7 from 32 plots had at least 5 years long census interval. The effect of CNDD was estimated by the Ricker model^7^. For individual species, it allows measuring overcompensating density dependence by computing the degree to which increasing conspecific adult densities decrease the recruitment of saplings, filtered from the effect of heterospecific densities. The Ricker model with a negative binomial error is fitted species by species in the form:

$$S_{i} = A_{i} \exp \left( {r + CNDD*A_{i} + HNDD_{adult} *a_{i} + HNDD_{sap} *s_{i} } \right)$$

$$S_{i} \sim {\text{NegBin}}\left( {S_{i},\gamma } \right)$$

where *S*_*i*_ is the observed number of saplings of the focal species in quadrat *i*, *A*_*i*_ is the number of conspecific adults of the focal species in quadrat *i*, *r* is the recruitment rate for the focal species at low conspecific adult densities, *CNDD* is the effect of conspecific adult density on sapling recruitment of the focal species, *HNDD*_*adult*_ is the effect of heterospecific adult density on sapling recruitment of the focal species, *a*_*i*_ is the observed number of heterospecific adults in quadrat *i*, *HNDD*_*sap*_ is the effect of heterospecific sapling density on sapling recruitment of the focal species, and *s*_*i*_ is the observed number of heterospecific saplings in quadrat *i*, and γ is the negative binomial overdispersion parameter for the focal species. Using the Ricker model, we isolated conspecific density effects (CNDD) relative to heterospecific effects (HNDD). Strong conspecific negative density dependence is reflected by low (or even negative) values of *CNDD* and weak or low negative density dependence corresponds to high values of *CNDD*. To calculate the strength of CNDD in each plot, the mean of *CNDD* values were computed across species in each plot. Following^7^, we excluded: 1) rarest species, of which adults or saplings occupied less than 10 quadrats and 2) species having their CNDD standard error above four from our analysis. For CNDD analysis, saplings were defined as trees having DBH smaller than 15 cm. The common threshold was chosen according to the size-classes distributions of species in the plots, to have enough species for the analyses. Originally, LaManna^7^ used up to 10 cm DBH trees recorded from 1 cm DBH. To allow the comparison of our and original results, we also provide some particular results for 10 cm sapling threshold. For example, just one tree species from 16 had reproductive size below 10 cm DBH in highly diverse tropical forest^47^, and by including trees with DBH ≥ 4.8 cm we may miss part of important density-dependent processes (i.e. the smallest size classes)^48^. If such threshold results in having less than 20% of individuals classified as adults, then 10 cm or, if this is still not enough, 5 cm are used as new thresholds. HNDD effects were weak (close to 0), therefore only CNDD results were presented (similar to^7^). Tree densities, measurement error, and dispersal rates in the forest plots may affect the estimation of CNDD, but simulation tests indicated that the above method is robust to these potential biases^7^.

### Environmental characteristics, CNDD and species diversity

Correlations between environmental characteristics, CNDD and species diversity were tested by generalized linear models for all plots and within forest types. Individual species analyses of CNDD considers just species occurring in at least six sites. To test the effects of interactions between environmental gradients and CNDD on species diversity, generalized linear models for species number with Poisson distribution were fitted. Specifically, the models with just additive effects (e.g. CNDD and altitude) were compared by Chi-square test with the models also having interaction terms (e.g. interaction between CNDD and altitude). If the latter model is better than the former, then the effect of CNDD on species diversity changes along the environmental gradient.

Regardless of forest type, we identify significant correlations between our environmental gradients: altitude and snow depth were negatively correlated and precipitation was positively correlated with temperature; snow depth was positively correlated with altitude (Fig. S1).

### Spatial aggregation

The most common reasons for aggregation of individuals are dispersal limitation and environmental heterogeneity^49^. To test the effect of small scale (as the size of quadrats) aggregation (clumping) of individual trees on CNDD (suggested by^12^), and its correlation with environmental characteristics and species diversity, individual species spatial patterns were described by an inhomogeneous pair correlation function (PCF). PCF allows to classify the distribution of individuals in space as aggregated (clumped), regular or random^50^. The strength of observed spatial patterns was compared with the 199 simulations of the inhomogeneous null model, which filters out the inhomogeneous density of individuals non-parametrically estimated by a Gaussian smoothing function^46^ with SD (bandwidth) of 30 m (as in similar plots^32^^.^). The density of individuals is commonly varying over the large scales in the plots (e.g. due to the habitat preferences of species on scales greater than 30–50 m). The inhomogeneous null model is filtering out these density patterns and therefore allows to reveal small scale species aggregation patterns mostly driven by interactions between trees (by so-called separation of scales)^46,50^. Individual species patterns were computed for each species having at least 25 individuals in the plot and their significance was gained by goodness-of-fit test^51^. Finally, the proportion of significantly aggregated species (P ≤ 0.05) was computed for each plot and its correlation to CNDD was analyzed.

All analyses were done in R 3.6.1^52^, the Ricker model was fitted by R package “gnm” using the R script provided in^7^. Spatial aggregation was computed in “spatstat” package^53^ and isotropic edge correction was applied to reduce the effect of individuals having low number of neighbours, because of being close to the border of plot^50^.

## Results

**Figure 2:**
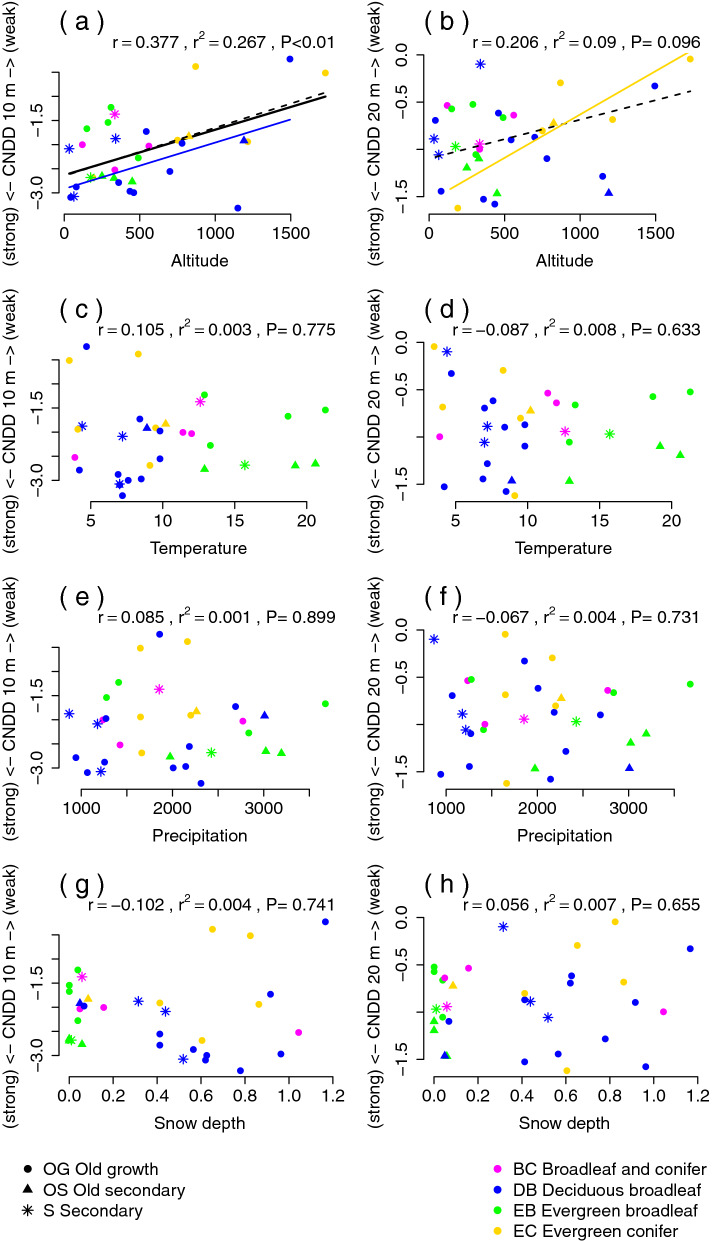
Correlations of conspecific negative density dependence (CNDD) with environmental characteristics for 10 m (**a**,**c**,**e**,**g**) and 20 m (**b**,**d**,**f**,**h**) spatial scales. Coloured points correspond to four forest functional types and point characters classify forests according succession stage. Coloured lines are shown if there was a significant linear correlation (P ≤ 0.05) inside the forest type, except black line that corresponds to an overall correlation without considering the forest types and succession stages. Solid lines are used for correlations of all forest succession stages and dashed lines represent correlation limited to old growth forests. Forest types are shown by colours: evergreen broad-leaved, deciduous broad-leaved, broad-leaved and conifer, and evergreen conifer. r is Spearman correlation coefficient, P is significance and r^2^ is a fraction of variance explained by the linear model of overall correlation (all types and succession stages of forests).

**Figure 3:**
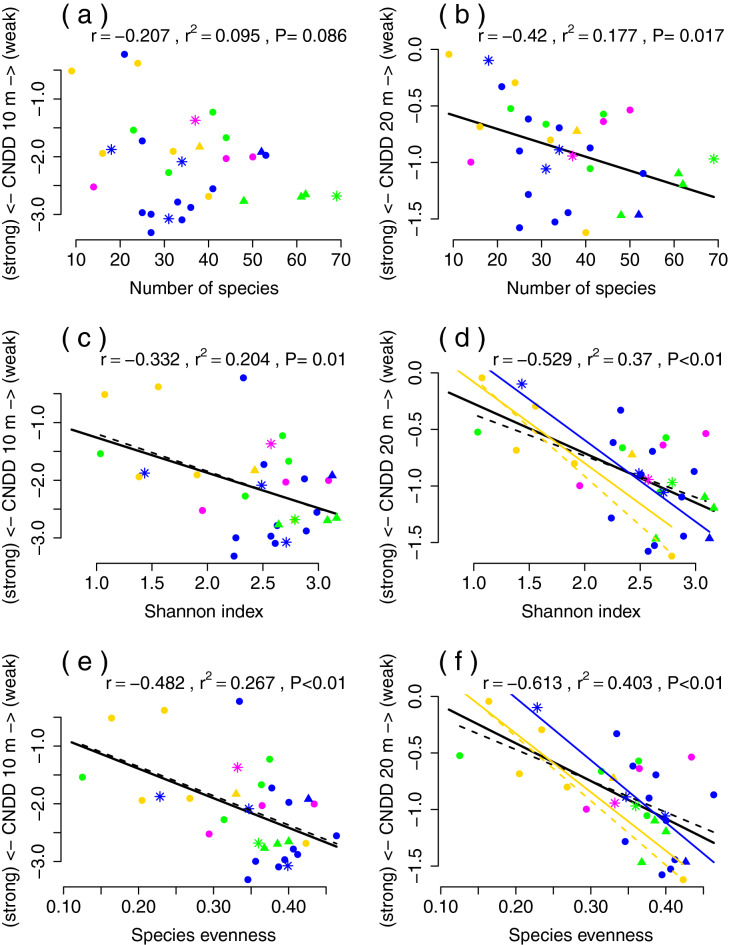
Correlations of CNDD with the species number, Shannon index and species evenness for 10 m (**a**,**c**,**e**,**g**) and 20 m (**b**,**d**,**f**,**h**) scales. Coloured points correspond to four forest functional types and point characters classify forests according succession stage. Coloured lines are shown if there was a significant linear correlation (P ≤ 0.05) inside the forest type, except black line that corresponds to an overall correlation without considering the forest types and succession stages. Solid lines are used for correlations of all forest succession stages and dashed lines represent correlation limited to old growth forests. Forest types are shown by colours: evergreen broad-leaved, deciduous broad-leaved, broad-leaved and conifer, and evergreen conifer. r is Spearman correlation coefficient, P is significance and r^2^ is a fraction of variance explained by the linear model of overall correlation (all types and succession stages of forests).

The strength of CNDD was negatively correlated with altitude in the 10 m and 20 m spatial scales for old-growth forests, and for all forests (including secondary forests) for the 10 m squares only (Fig. 2a,b). At the lower altitudes, there was stronger CNDD than at the higher altitudes. The same correlations between CNDD and altitude were also found for some forest types (e.g. within deciduous broadleaf and evergreen conifer forests). No significant correlations of CNDD with temperature, precipitation and snow depth were identified (Fig. 2). CNDD was posi﻿tively correlated with most of diversity indices (number of species, Shannon index and species evenness) for all forests in both spatial scales; CNDD tended to be strongest in the most diverse forests (Fig. 3). Using 10 cm sapling threshold leads mostly to the same diversity indices correlations (compare Fig. 3 and Fig. S2). The results of the CNDD and heterospecific negative density dependence (HNDD) were the same as CNDD only, because the effects of HNDD were weak, mostly none (Fig. S3). No differences were observed in the strength of CNDD between forest types and succession stage categories (Fig. S4). Individual species CNDD did not follow the overall results (Table 1). A maximum of three species from nine exhibited significant correlations between CNDD and the number of species or species evenness at some of the spatial scales. But individual species CNDD was negatively correlated with their log-transformed basal areas; more common species had weaker CNDD than less common ones (Fig. S5).

**Table 1:** Correlations of individual species CNDD values with environmental gradients (altitude, temperature, precipitation and snow depth) and diversity characteristics.

Species	n	Altitude	Temperature	Precipitation	Snow depth	Number of species	Shannon index	Species evenness
*Camellia japonica*	8	−, −	−, +	−, −	+ , −	+ , +	−, −	−, +
*Cleyera japonica*	8	+ , +	−, −	−, −	+ , +	−*, −*	−, −	−, −
*Eurya japonica*	8	+ , +	−, −	−, +	+ , +	−, +	−, −	−*, −
*Distylium racemosum*	6	−, −	−, +	+ , +	−, −	−, +	−, −	−, −
*Acer japonicum*	7	−, +	−, −	+ , −	+ , +	−, −	−, −^x^	−, −*
*Magnolia obvata*	7	+ , −	+ , −	+ , −	+ , + *	−*, −*	−, −^x^	−, −
*Quercus salicina*	7	−, −	+ , +	+ , −	−, −	+ , +	+ , + *	+ , + *
*Quercus crispula*	8	−, −^x^	−, −	−, −^x^	−, +	−, −^x^	−, −	−, −
*Fagus crenata*	9	+ , + ^x^	−*, −	−, −	+ *, −	−*, +	−, −	−, −

The signs of correlations (+ for positive correlation and − for negative one) are shown for 10 m scale (before comma) and for 20 m scale (after comma). n is number of plots where species occur, * denotes significant correlations with P ≤ 0.05 and ^x^ is for P ≤ 0.1.

All diversity indices were negative correlated with altitude for old-growth forests and evergreen forests (Fig. S6). Temperature and precipitation were positively and snow depth negatively correlated with the number of species for all forests. The Shannon index and species evenness were positively correlated with temperature just for deciduous broad-leaved forests. Similarly, the Shannon index was negatively correlated with snow depth for all forests.

**Figure 4:**
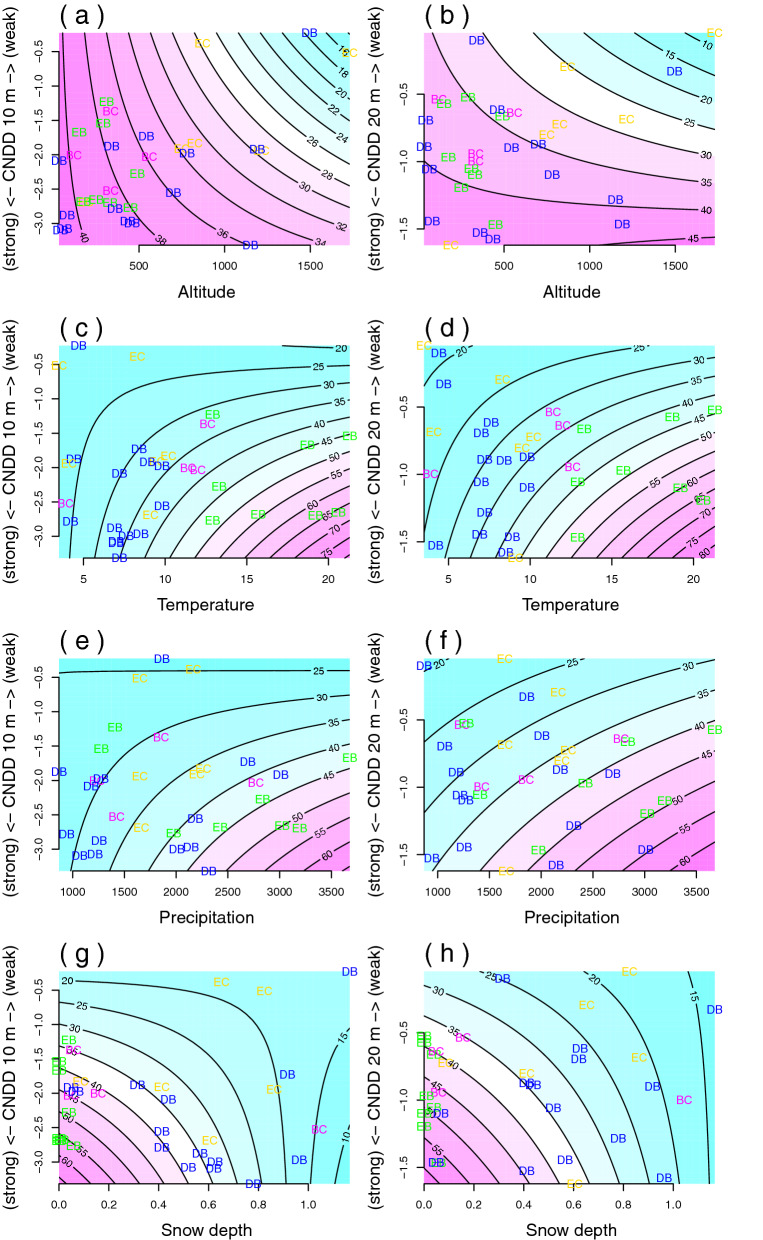
Contour plots from linear models explaining species number by CNDD in 10 m (**a**,**c**,**e**,**g**) and 20 m (**b**,**d**,**f**,**h**) spatial scale across environmental gradients of altitude, temperature, precipitation and snow depth. Contour lines and colours describe predicted species number. Observed values are shown by characters corresponding forest types.

CNDD and environmental gradients alone had significantly explained the species number (individual additive effects; Fig. 3, Fig. S6). In addition, their interactions had significant effects on species number, specifically in cases of altitude, temperature and snow depth as environmental gradients (Table 2). The best predictors of species number were CNDD with snow depth and temperature with 50–72% explained variability (considering both spatial scales). Significant interactions reflect that CNDD effects on species number are changing along environmental gradients (Fig. 4). The highest species number is found under the highest CNDD and low altitudes, high temperature, high precipitation and low snow depth. In contrast, with low temperature, low precipitation and high snow depth, the changes of CNDD had a weak effect on the species number. The relationship with altitude was more complex, the highest species numbers were not at the highest values of CNDD (Fig. S7). The same results were observed even if we included the number of trees as a predictor of species number in the models. No significant effects of interactions of CNDD and precipitation on species number were observed (Table 2).

**Table 2:** Number of species explained by generalized linear models across environmental gradients (altitude, temperature, precipitation and snow depth referred in models as ENV) and CNDD in 10 m and 20 m scale.

Environmental gradient (ENV)	Scale	Species number (estimate)—full model				Explained variance (R^2^)		
		Intercept	ENV	CNDD	Interaction of ENV with CNDD	ENV	ENV and CNDD (no interaction)	ENV and CNDD with interaction
Altitude	10 m	3.80	− 6.47e−04*	3.21e−02^X^	− 1.76e−04*	15.00%	16.95%	19.50%
	20 m	3.72	− 8.18e−04*	0.82e−02*	− 6.20e−04*		24.99%	32.62%
Temperature	10 m	3.03	0.01*	− 0.01*	− 0.02*	38.89%	47.98%	50.40%
	20 m	2.86	0.03*	− 0.18*	− 0.03^X^		54.96%	56.48%
Precipitation	10 m	3.16	1.06e−05*	1.12e−02*	− 9.61e−05	15.89%	26.21%	27.07%
	20 m	2.84	1.81e−04*	2.28e−01*	− 3.30e−05		31.13%	31.18%
Snow depth	10 m	3.08	− 0.23*	− 0.37*	0.33*	54.91%	65.93%	70.14%
	20 m	3.36	− 0.59*	− 0.56*	0.31		70.33%	71.26%

Estimated coefficients are shown with significance (* for P ≤ 0.05, ^x^ for P ≤ 0.1) and explained variance is in %, both for 10 m and 20 m quadrats. The coefficient of determination (R^2^) in generalized linear models was computed gradually (only ENV, ENV and CNDD, ENV * CNDD) as 1-residual deviance over null deviance.

The proportion of aggregated species was not correlated with CNDD in the 10 m and 20 m spatial scales, environmental characteristics and species diversity, except for correlation with precipitation and snow cover for deciduous broadleaf forests (Fig. S8).

## Discussion

Our results support the hypothesis that CNDD changes across wide environmental gradients^6,20^, in particular, the strength of CNDD was observed to decrease towards high altitudes. Moreover, strong CNDD is correlated with high species richness and diversity (species evenness was the most tightly correlated with CNDD; Fig. 3). The strong effect of conspecifics on local survival in high diversity forests supports predictions of the Janzen-Connell hypothesis that CNDD is a process expected to maintain high species diversity^3,4,54^. Moreover, the combined effects of CNDD and environmental gradients on species diversity were observed, definitely the most strongly for snow depth, but also for altitude and temperature.

Changes in the strength of CNDD may be driven by underlying environmental conditions. Recent studies suggested that CNDD increases with precipitation and productivity at continental to global scales^6,20^. It was hypothesized that this relationship is due to the stronger effect of desiccation-intolerant natural enemies and pathogens (e.g. fungi and insects) in wetter sites^20,55^. Moreover, stronger intraspecific competition and stronger host-specific antagonistic interactions (e.g. due to soil pathogens and plant herbivores) in more resource-rich environments might cause stronger CNDD^27^. The same processes can change the strength of CNDD across other environmental gradients. We observed a negative correlation of CNDD with altitude (the strongest CNDD was in the lowest altitudes), but there was no significant relationship with precipitation, temperature or snow depth. This can be caused by the less precipitation but large temperature gradient in Japan (mesic to humid vs. subarctic to subtropical climate), where species distribution is not limited by precipitation, but it is mainly controlled by temperature gradient^35,36^. Altitude was not linearly correlated with precipitation (the relationship was rather unimodal), but it is tightly correlated with temperature and snow depth. CNDD correlation with altitude is expected to be due to a compound effect of altitude, that consists of several underlying environmental gradients, similar to latitudinal gradient^26^. Thus, species distributions are not related to individual gradients, but reflect the complexity of several environmental gradients. Moreover, herbivore and pathogen attacks respond more strongly to seasonality (e.g. length of growing season) than to mean climate values^30^ and changes in CNDD across resource gradient may differ among common and rare species, and life stages^27^.

Strong CNDD may prevent erosion of biodiversity in the forests by limiting populations of common species and more strongly stabilizing the populations of rare species^7^. Accordingly, we observed that the strength of CNDD was negatively correlated with species basal areas and positively correlated with species diversity, even within forest types. This directly supports the Janzen-Connell hypothesis stating CNDD as the key process responsible for reduced recruitment near conspecific adults and creating space available for other species^3,4^. Moreover, this suggests that local biotic interactions are underlying species richness, although they are also affected by climatic gradients^24^. To explain species number in the forests, several environmental gradients were interacting with CNDD. In particular, snow depth, temperature and altitude were the best predictors of tree species number. Similarly, temperature affected the overall beta diversity of tree species and elevation differences had a strong effect on the beta diversity of conifers in Japan^33^. Although species number was observed to increase with precipitation, we did not find a significant interaction of CNDD with precipitation affecting the species number. Surprisingly, we observed that snow depth was the best predictor for species diversity, even stronger than temperature. Hence, we suggest that species diversity may be also limited by individual species tolerance to snow damage in Japan^40,41^. Experimental tests, however, are needed to determine the relative importance of CNDD across environmental gradients.

Evolutional history, space environmental limitations (e.g. by climate) and local biotic interactions are known drivers of global patterns of tree species diversity^16,19^. These characteristics restrict how many species can survive at a given location as a result of physiological species limits and evolutional history^23^. Environmental conditions then directly favour species with suitable niche ranges or through the limiting number of individuals (carrying capacity) moderated by productivity^25,56^. In the Japanese archipelago, the beta diversity patterns of woody plants are formed by the combination of geographical isolation, the increasing frequency of typhoons and environmental filtering as responses to geohistorical perturbations and environmental gradients^32,33,57,58^. Thus, there is a strong unique regional effect on patterns of species diversity^19^.

We found support for species richness patterns driven by local biotic interactions; their strength was changing along environmental gradients. Our results suggest that CNDD is an important mechanism structuring forest communities across several environmental gradients. We found that the strength of CNDD was observed to be an important factor for species diversity in low altitudes with high temperatures and low snow depth. The less climatically or environmentally limited habitats are expected to be under stronger effects of biotic interactions, such as competition or pressure from specialized enemies, in the community assembly^27,59^. In contrast, CNDD was weakest in high altitudes and diversity was lowest in these habitats. Species from high altitudes should have some physiological tolerance for such environmentally-limited habitats^26,59^. The strength of CNDD of individual species in multiple plots mostly did not change along the gradients, therefore species turnover is expected to be responsible for differences in overall correlations of CNDD across environmental gradients. This is concordant with a shift in the assembly process from stochasticity (driven by selection effect) in productive sites to deterministic assembly (due to species niche differences) in the harsh environments of the Japanese forests^37^.

There are a few caveats in our study. Many ecological processes ongoing in the forest directly affect diversity patterns, e.g. small-scale environmental heterogeneity due to topography (not recorded by Monitoring Sites 1000 Project) drives the distribution of individual species^46,60,61^, which could affect CNDD via the distribution of individual trees. For example, intraspecific aggregation caused density-dependent mortality in Japanese forests^34^. Similarly, CNDD patterns along the gradients^7^ was interpreted as results of individual aggregation^12^, which is weaker for species with high CNDD and can be induced by other underlying processes, including dispersal limitation and environmental heterogeneity^62^. However, additional methods supporting the original observations of CNDD patterns were presented^13–15^ and pointed out several statistical and conceptual problems of the methods in the comments^10,12,62^ to the original study (e.g. Detto’s study^12^ conclusions are based on single iteration of not appropriate null model). In particular, we did not observe any strong aggregation patterns in our data by comparing individual species spatial patterns (under the inhomogeneous null model that controls for species and forest specific density patterns) and CNDD, environmental characteristics or species diversity. Still, because of stochastic dilution problem (due to errors in explained variables) and analyses of single census (static data) for studying CNDD^62,63^, our results should be interpreted with caution, even through all results along the gradients should be affected similarly. Generally, the effect of CNDD is the strongest for the smallest trees^20,29^, although trees with DBH ≥ 4.8 cm were recorded and analysed in our dataset. On the other hand, by not including the smallest trees we avoid the effect of overgrazing by deer, which has been a major problem preventing tree recruitment in Japan, especially in recent years^64^.

## Conclusions

We found significant effects of local conspecific density on diversity in plant communities along four environmental gradients in the Japanese archipelago. The strength of local conspecific negative density dependence (CNDD) changes with altitude, and temperature and snow depth, which also affect species number. All these environmental gradients could be considered as surrogates of anthropogenic climate change^65^. Regardless of the mechanisms involved, our results suggest that along the gradients, biotic interactions (represented by CNDD) were changing and have strong effects on species composition and diversity in forests. These effects are important under the current climate change scenario, especially when: (1) most of the endemic species in Japan are locally restricted in the mountain areas and the archipelago provides a regional diversity hotspot^57^, (2) some forest types are at the edges of their climatic ranges and regulated by temperature^33^, (3) we observed that species diversity was highly correlated with altitude, temperature and snow depth gradients.

## Supplementary Information

Supplementary Information.

## Data Availability

All data are deposited in data paper (see^45^) and free available from http://db.cger.nies.go.jp/JaLTER/ER_DataPapers/archives/2011/ERDP-2011-01/.
