# Author Correction: Contribution of conspecific negative density dependence to species diversity is increasing towards low environmental limitation in Japanese forests

**DOI:** 10.1038/s41598-021-04348-8

**Published:** 2021-12-23

**Authors:** Pavel Fibich, Masae I. Ishihara, Satoshi N. Suzuki, Jiří Doležal, Jan Altman

**Affiliations:** 1 Institute of Botany, Czech Academy of Sciences, Průhonice, Czech Republic; 2 Department of Botany, Faculty of Science, University of South Bohemia, České Budějovice, Czech Republic; 3 Field Science Education and Research Center, Ashiu Forest Research Station, Kyoto University, Kyoto, Japan; 4 Graduate School of Agricultural and Life Sciences, The University of Tokyo Hokkaido Forest, The University of Tokyo, Furano, Japan

Correction to: *Scientific Reports* https://doi.org/10.1038/s41598-021-98025-5, published online 21 September 2021

The Supplementary Information file published with this Article contained an error where the last four rows of data in Supplementary Table S1 were omitted. The original Supplementary Information file is provided below.

This error has now been corrected in the Supplementary Information file that accompanies the original Article.

## Supplementary Information

Supplementary Information.

