# Supplementary material for: Contribution of conspecific negative density dependence to species diversity is increasing towards low environmental limitation in Japanese forests
Source: Sci Rep. 2021 Sep 21;11:18712. doi: 10.1038/s41598-021-98025-5 (PMC8455644; doi:10.1038/s41598-021-98025-5)
Supplement: Supplementary file 1 — Supplementary Information. [file 41598_2021_98025_MOESM1_ESM.pdf]

Supplementary materials of “Contribution of conspecific negative density dependence to species diversity is increasing towards low environmental limitation in Japanese forests” by Fibich P, Ishihara MI, Suzuki SN, Dolezal J, Altman J.

Table S1. Forest plot characteristics. Temperature was corrected for altitudinal difference between the plots and the 1 km grid mean by the lapse rate of 0.6°C per 100m (Barry, 2008). We did not use rarefied diversity indices for the common plot size as the results were the same for non-rarefied diversity. All data are deposited in a data paper (see Ishihara et al., 2011).

PlotID	SiteName	Type	Status	Latitude	Longitude	Altitude	Area	Temperature	Precipitation	Show depth	#trees	#species	Shannon	Evenness
**AI-BC1**	Aichi-akazu	BC	S	35.22	137.17	335	1	12.6	1854.3	0.06	2322	37	2.57	0.33
**AM-EB1**	Amami	EB	OS	28.33	129.45	330	1	19.2	3193.8	0	2984	61	3.08	0.39
**AO-BC1**	Aobayama	BC	OG	38.25	140.85	120	1	11.4	1236.1	0.17	1239	50	3.09	0.43
**AS-DB1**	Ashoro	DB	OG	43.32	143.51	360	1	4.2	942.1	0.51	643	33	2.63	0.41
**AS-DB2**	Ashoro	DB	S	43.26	143.51	340	1	4.4	867.9	0.37	530	18	1.43	0.23
**AU-DB1**	Ashiu	DB	OG	35.35	135.74	700	1	9.8	2184.5	0.51	625	41	2.99	0.46
**AU-EC1**	Ashiu	EC	OG	35.35	135.74	750	1	9.5	2199.8	0.51	1215	32	1.91	0.27
**AY-EB1**	Aya	EB	OG	32.05	131.19	490	1	13.3	2835.6	0.04	1727	31	2.34	0.31
**GR-DB1**	Mt. Garyu	DB	OG	34.69	132.19	1150	1	7.2	2313.6	1.18	652	27	2.24	0.35
**HY-EC1**	Hayachine	EC	OG	39.54	141.5	1215	1	4.1	1649.6	1.37	859	16	1.38	0.2
**IC-BC1**	Ichinomata	BC	OG	33.15	132.92	560	0.95	12	2770.8	0.05	1674	44	2.71	0.36
**KA-EB1**	Kasugayama	EB	OG	34.68	135.86	310	1	12.9	1408.8	0.04	1268	41	2.68	0.37
**KJ-EB1**	Kasuya	EB	OS	33.65	130.55	450	1	12.9	1974.1	0.06	1317	48	2.64	0.37
**KK-DB1**	Kanamegawa	DB	OG	38.15	139.84	543	1	8.4	2691.2	1.5	771	25	2.51	0.38
**KM-DB1**	Kanumazawa	DB	OG	39.11	140.86	435	1	8.5	2144	1.62	674	25	2.57	0.39
**KY-DB1**	Kayanodaira	DB	OG	36.84	138.5	1495	1	4.7	1860.3	2.21	1045	21	2.32	0.33
**NB-EC1**	Nibuna-mizusawa	EC	OG	40.08	140.25	190	1	9.1	1663.5	0.83	720	40	2.78	0.42
**NP-DB1**	Nopporo	DB	OG	43.06	141.53	42	1.04	7	1066.8	0.86	853	34	2.61	0.39
**OS-EC1**	Osado	EC	OG	38.21	138.44	870	1	8.3	2164.4	0.92	764	24	1.56	0.23
**OT-EC1**	Otanomosutaira	EC	OG	36.7	138.5	1730	1	3.5	1648.2	1.28	701	9	1.07	0.16
**OW-EB1**	Ogasawara-sekimon	EB	OG	26.68	142.16	290	1	21.3	1276.7	0	3880	23	1.04	0.13
**OZ-DB1**	Otakizawa	DB	OG	39.64	140.89	460	1	7.6	2006.6	0.87	565	27	2.26	0.36
**SI-DB1**	Shiiba	DB	OS	32.38	131.1	1190	1	8.9	3009.4	0.05	1520	52	3.12	0.43
**TB-DB1**	Mt. Tsukuba	DB	OG	36.23	140.1	780	1	9.8	1271	0.07	1313	53	2.87	0.4
**TM-DB1**	Tomakomai	DB	OG	42.71	141.57	80	1	6.9	1255.4	0.76	1113	36	2.89	0.41
**TM-DB2**	Tomakomai	DB	S	42.69	141.59	64	1.2	7	1215.5	0.68	892	31	2.71	0.4
**TM-DB3**	Tomakomai	DB	S	42.67	141.63	33	0.81	7.2	1178.3	0.55	1303	34	2.49	0.35
**TN-EB1**	Tano	EB	S	31.86	131.3	175	1	15.7	2426.2	0.01	2323	69	2.79	0.36
**UR-BC1**	Uryu	BC	OG	44.37	142.28	335	1.05	3.9	1424.2	1.84	770	14	1.95	0.29
**WK-EC1**	Wakayama	EC	OS	34.07	135.53	825	1	10.2	2262.2	0.09	1555	38	2.43	0.33
**YK-EB1**	Yakushima-shoyojyurin	EB	OG	30.37	130.39	150	1	18.7	3677	0	1828	44	2.73	0.36
**YN-EB1**	Yona	EB	OS	26.74	128.23	250	1	20.6	3023.1	0	2714	62	3.16	0.4

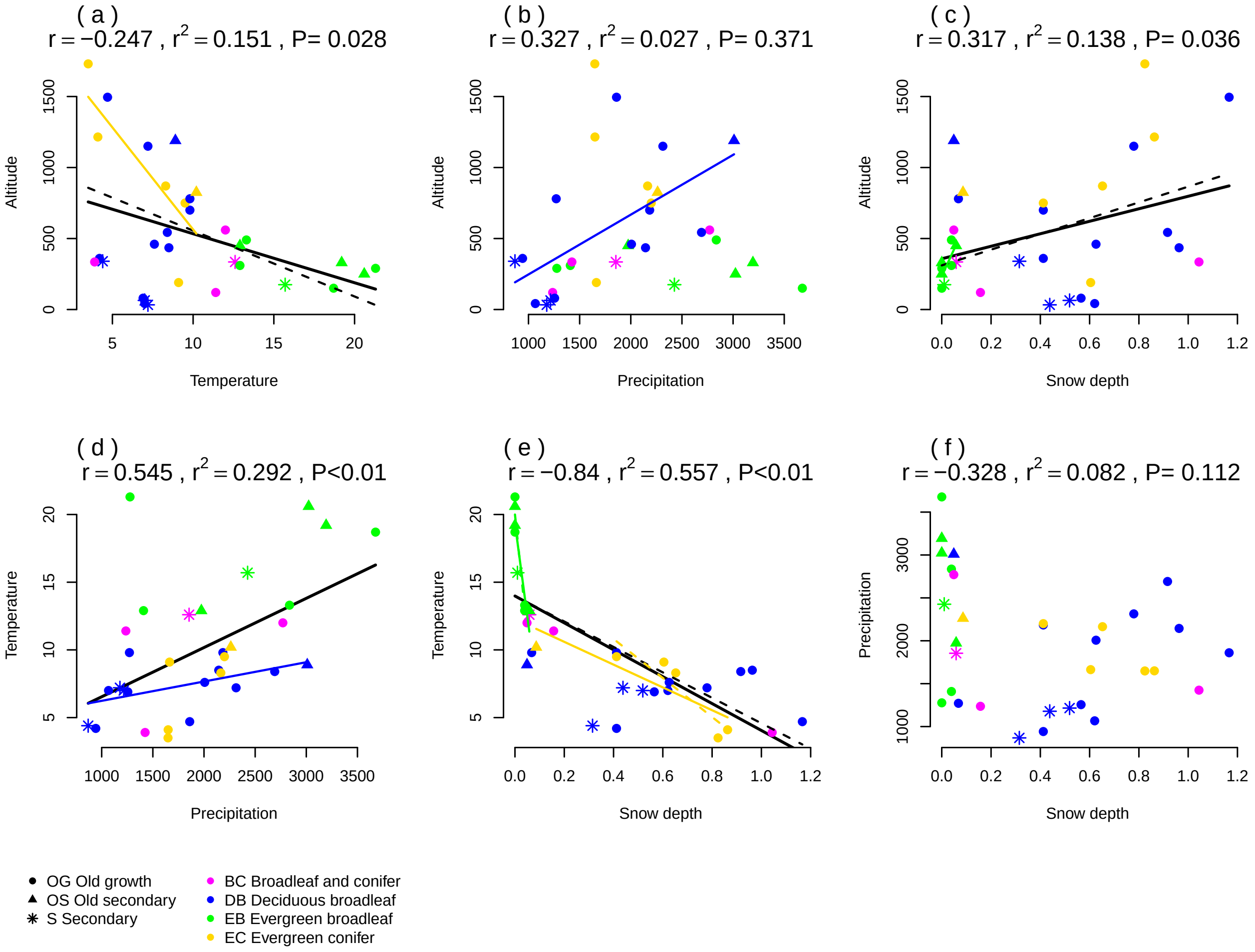

Figure S1. Correlations between altitude, temperature, precipitation and snow depth. Coloured points correspond to four forest functional types and point characters classify forests according succession stage. Coloured lines are shown if there was a significant linear correlation ($P \leq 0.05$) inside the forest type, except black line that corresponds to an overall correlation without considering the forest types and succession stages. Solid lines are used for correlations of all forest succession stages and dashed lines represent correlation limited to old growth forests. Forest types are shown by colours: evergreen broad-leaved, deciduous broad-leaved, broad-leaved and conifer, and evergreen conifer. r is Spearman correlation coefficient, P is significance and r^2 is a fraction of variance explained by the linear model of overall correlation (all types and succession stages of forests).

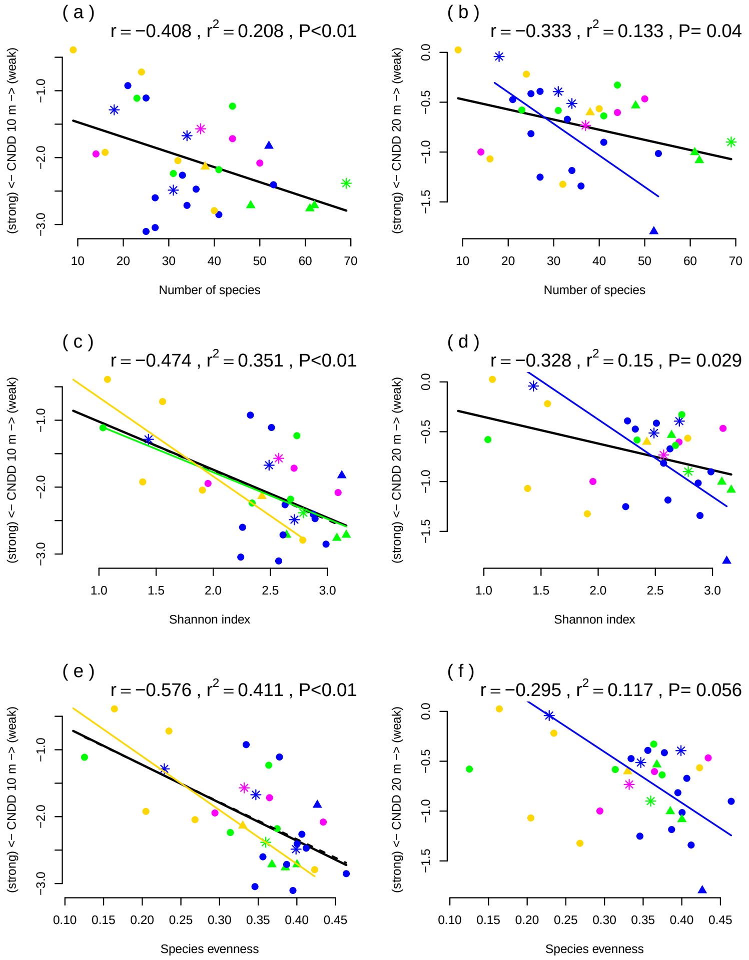

Figure S2. Correlations of CNDD with the species number, Shannon index and species evenness for 10 m (a, c, e, g) and 20 m (b, d, f, h) scales based on 10 cm DBH threshold for saplings. For the legend see **Fig. S1**.

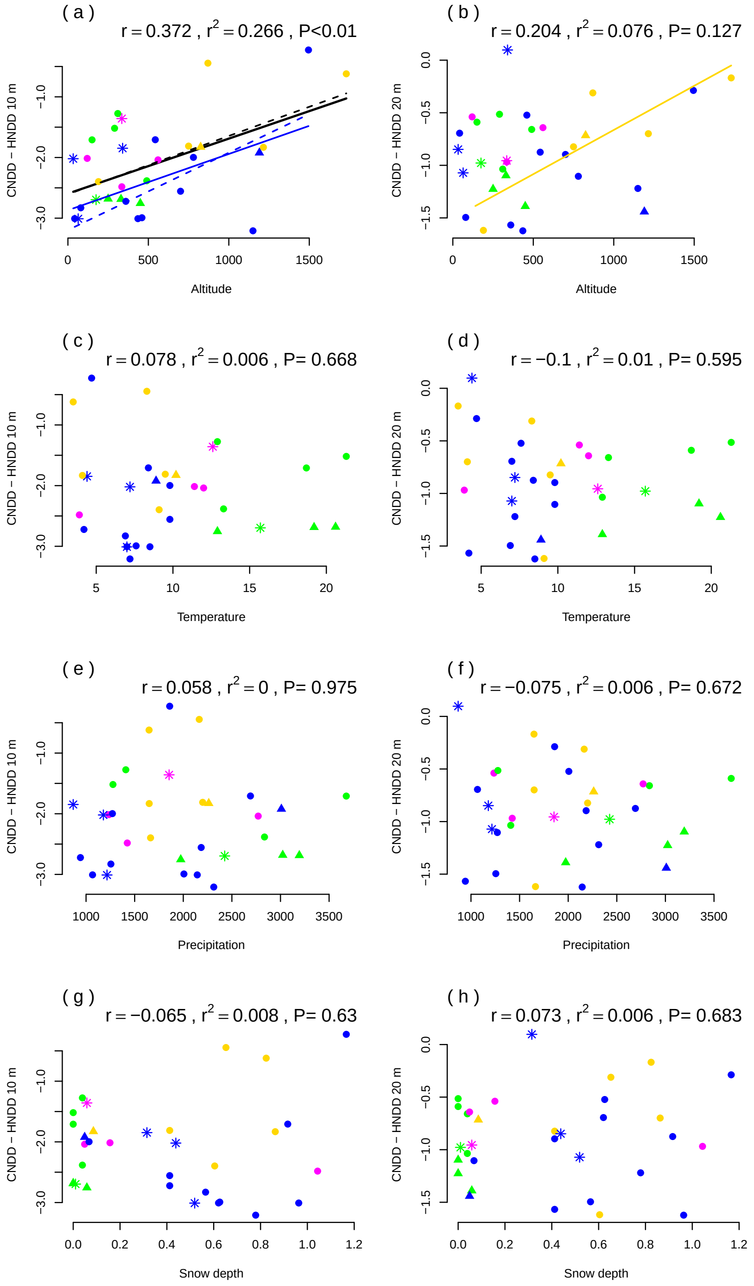

Figure S3. Correlations of conspecific negative density dependence (CNDD) minus heterospecific negative density dependence of adults (HNDDad) with environmental characteristics for 10 m (a,c,e,g) and 20 m (b,d,f,h) scales. For the legend see **Fig. S1**.

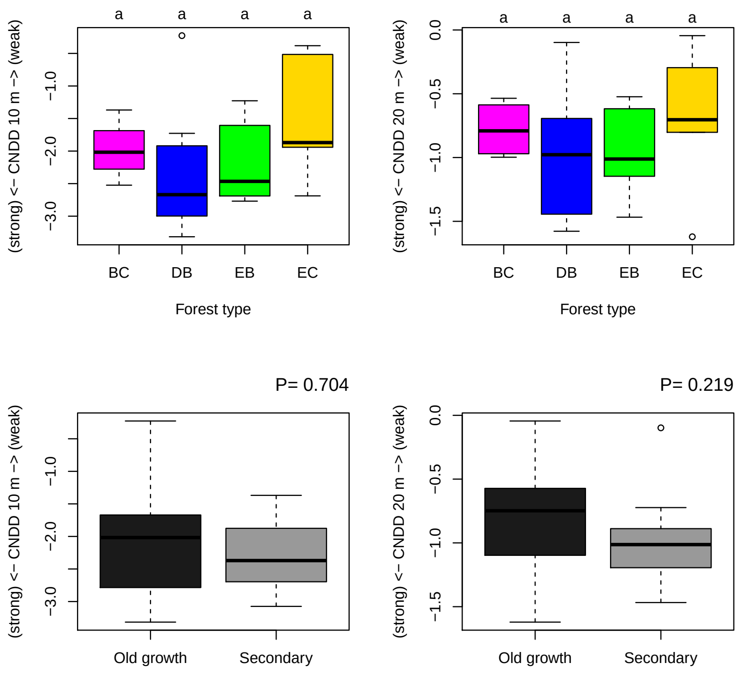

Figure S4. CNDD for the spatial scales, forest types and succession stage categories. Forest types: evergreen broad-leaved (EB), deciduous broad-leaved (DB), broad-leaved and conifer (BC) and evergreen conifer (EC). Wilcox test was used to compare pair of forest. a denote that there were no significant differences between forest types.

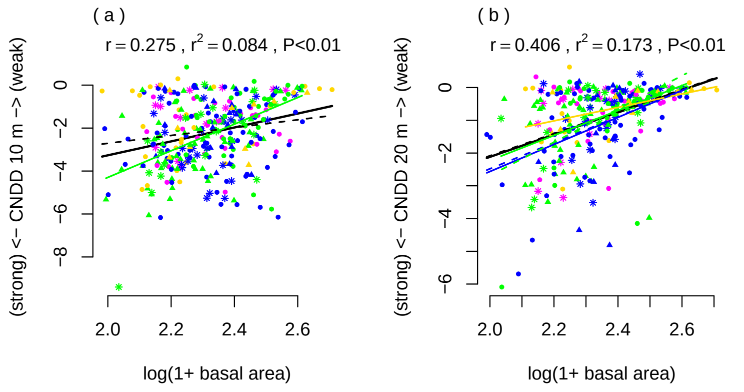

Figure S5. Correlations of conspecific negative density dependence (CNDD) of individual species with their log transformed basal areas for 10 m (a) and 20 m (b) scales. For the legend see **Fig. S1**.

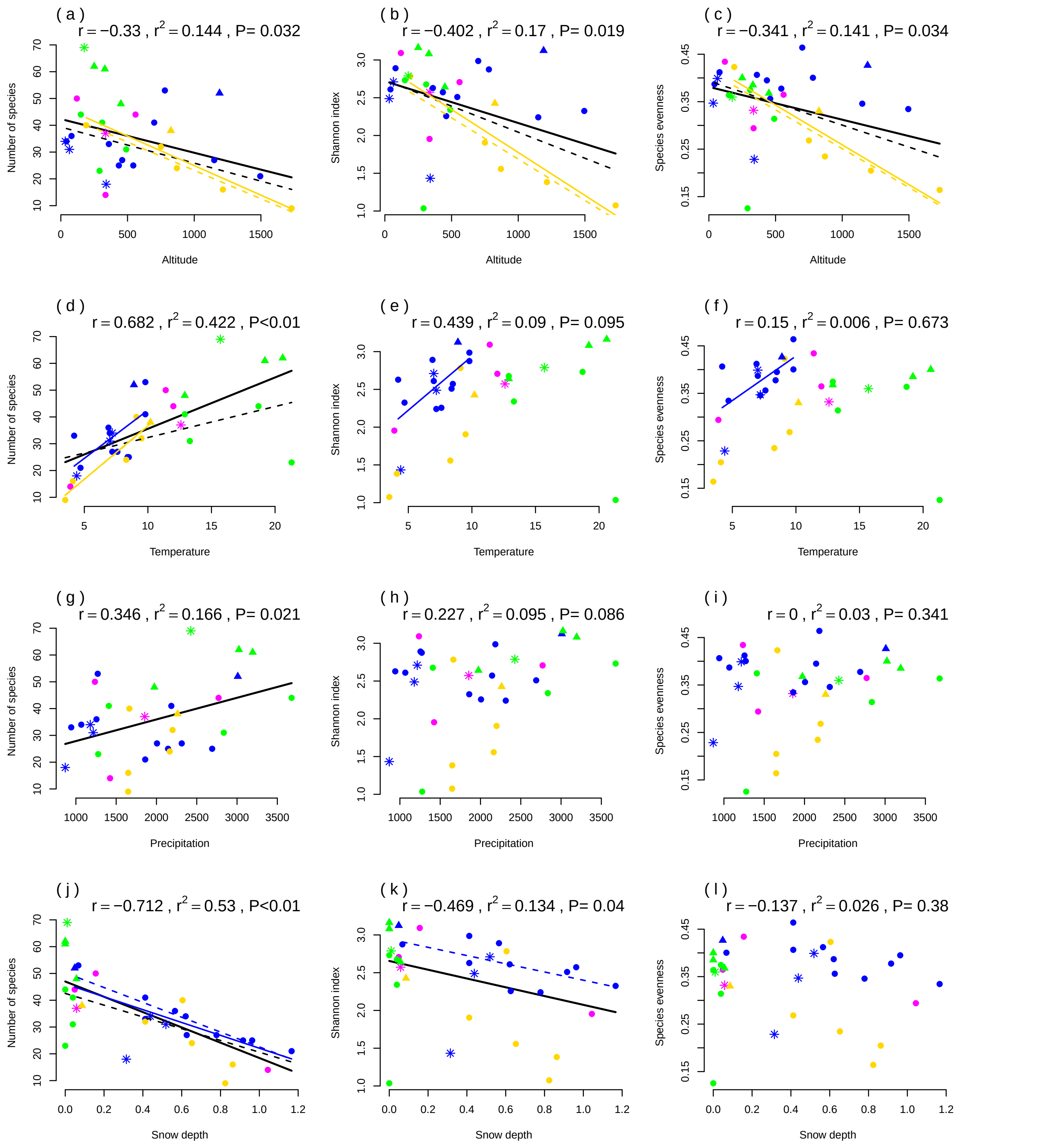

Figure S6. Correlations of diversity indices (number of species, Shannon index and species evenness) with altitude, temperature, precipitation and snow depth. For the legend see **Fig. S1**.

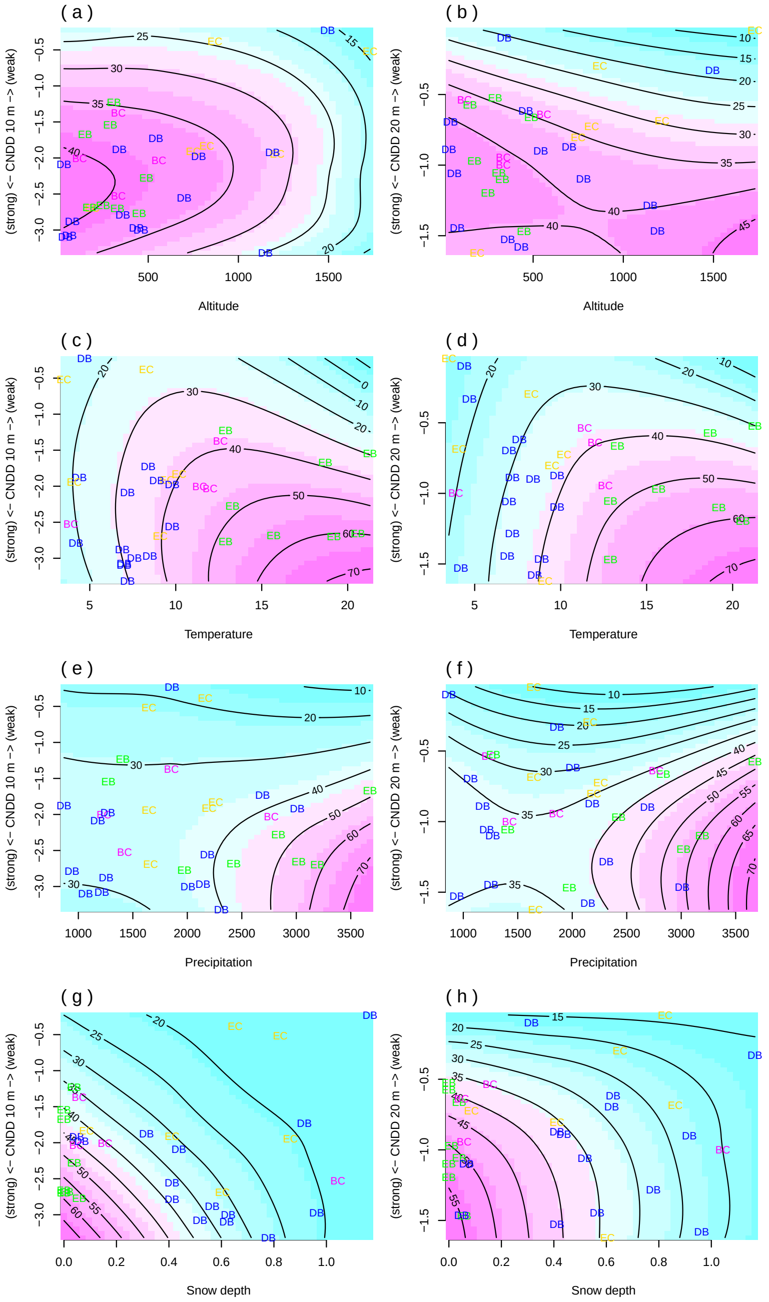

Figure S7. Contour plots from loess models explaining species number by CNDD in 10 m and 20 m spatial scale across environmental gradients of altitude, temperature, precipitation and snow depth. Contour lines and colours describe predicted species number. Observed values are shown by characters corresponding forest types. Loess was computed with 1.5 degree of smoothing (alpha).

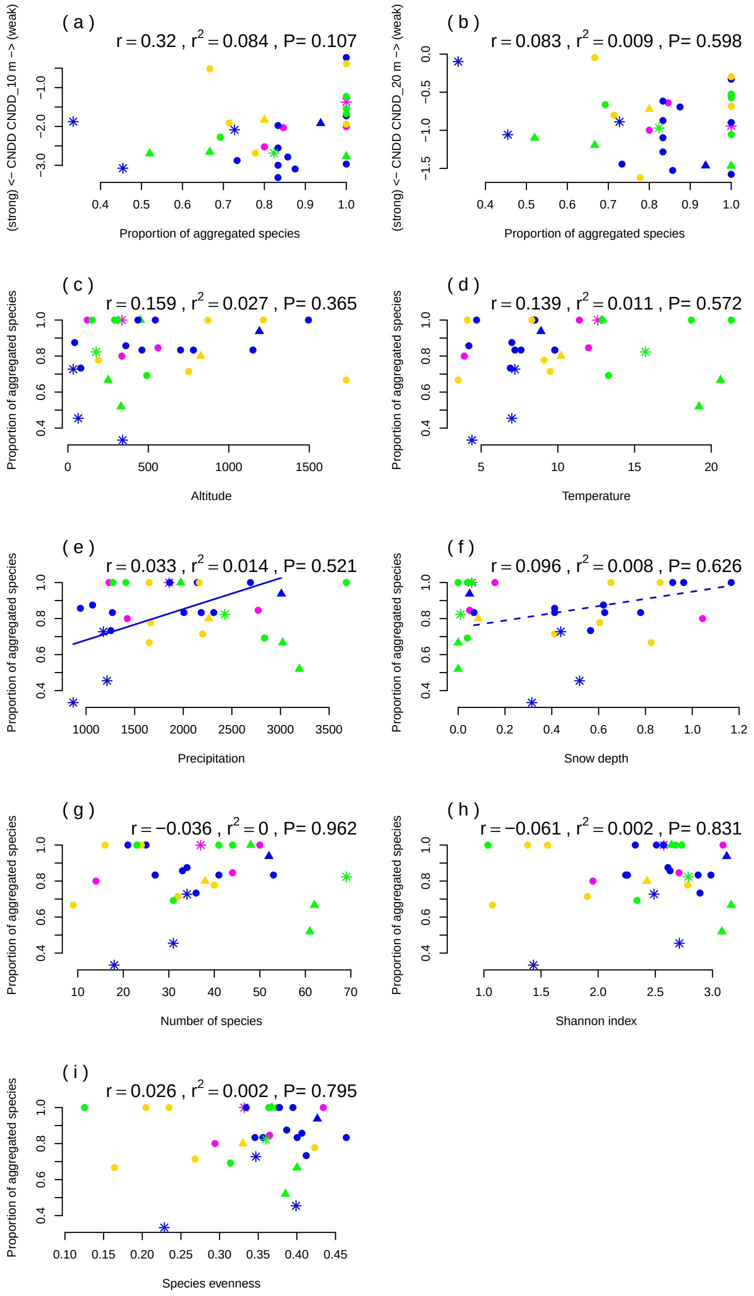

Figure S8. Correlations of the proportion of aggregated species (significant aggregation up to 10m by inhomogeneous pair correlation function) with CNDD (10 m and 20 m spatial scale), environmental characteristics (altitude, temperature, precipitation and snow depth) and diversity indices (number of species, Shannon index and species evenness). For the legend see **Fig. S1**.
